# Corrigendum: Microclimate shapes the phylosymbiosis of rodent gut microbiota in Jordan's Great Rift Valley

**DOI:** 10.3389/fmicb.2025.1639190

**Published:** 2025-06-18

**Authors:** Enas Al-khlifeh, Sanaz Khadem, Bela Hausmann, David Berry

**Affiliations:** ^1^Laboratory of Immunology, Department of Medical Laboratory Science, Al-Balqa Applied University, Al-Salt, Jordan; ^2^Division of Microbial Ecology, Department of Microbiology and Ecosystem Science, Centre for Microbiology and Environmental Systems Science, University of Vienna, Vienna, Austria; ^3^Joint Microbiome Facility of the Medical University of Vienna and the University of Vienna, Vienna, Austria; ^4^Division of Clinical Microbiology, Department of Laboratory Medicine, Medical University of Vienna, Vienna, Austria

**Keywords:** bioclimatic zone, gut microbiota, host phylogeny, microbiome, phylosymbiosis

In the published article, there was an error in Figure 3C as published. “Inverse Simpson Index” plot is a duplication of the “ASV richness” plot. The corrected Figure 3 and its caption appear below.

**Figure 3:**
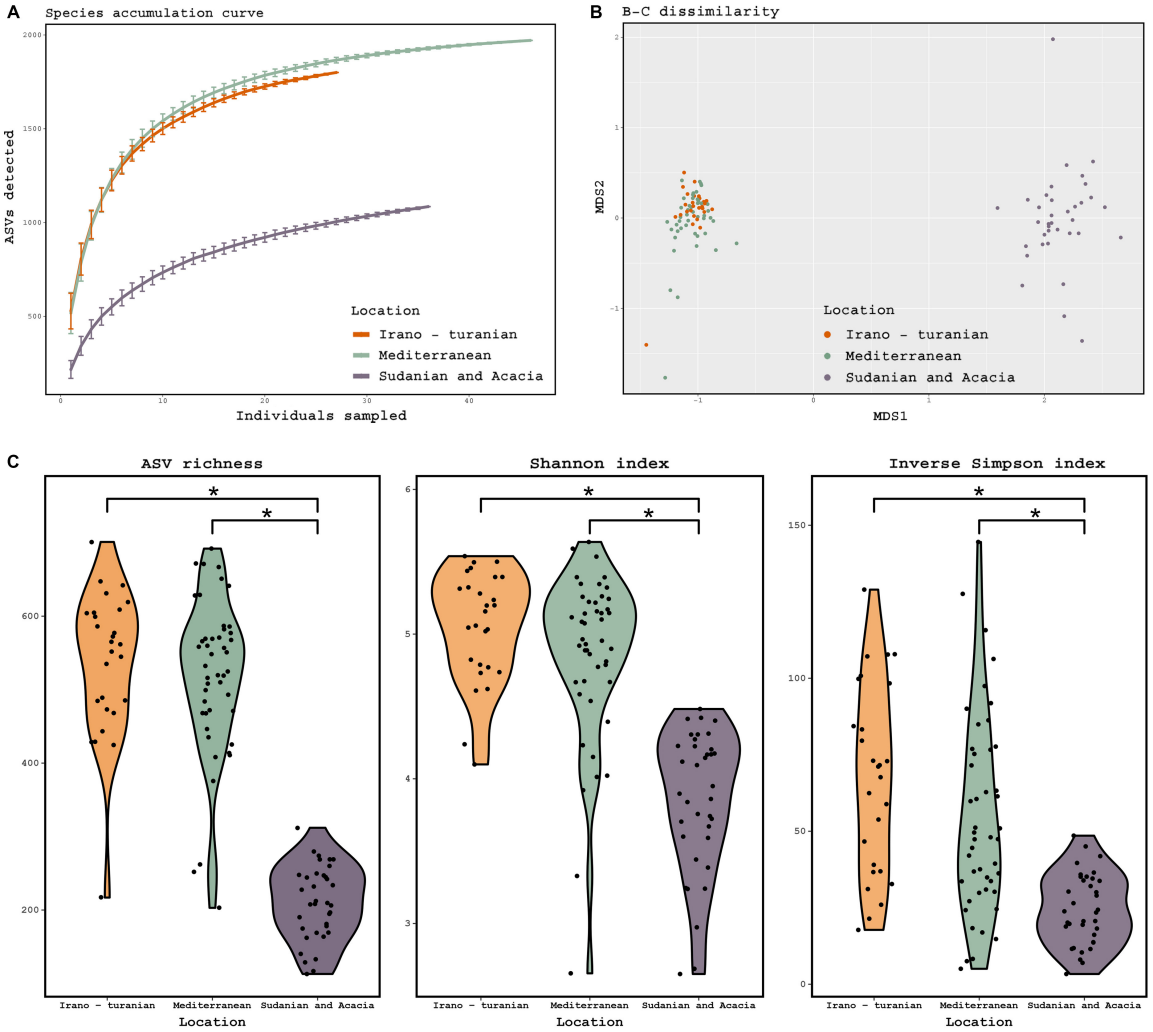
Diversity of the gut microbiome based on 16S rRNA gene amplicon analysis. **(A)** Species accumulation curve, determined at the level of ASVs. **(B)** Principal coordinate analysis ordinations based on Bray–Curtis distances of the gut microbiota of rodents collected from three bioclimatic zone, indicated by different colored dots. **(C)** Observed ASV richness, Shannon diversity, and inverse Simpson diversity across bioclimate zones. (ANOVA, *p* = 0.001; Tukey-HSD, *p* < 0.001). The threshold for significance is *p* = 0.05. The figure shows a black dot for the mean value and a colored region for the median value.

The authors apologize for this error and state that this does not change the scientific conclusions of the article in any way. The original article has been updated.

